# Spontaneous Pneumomediastinum and Subcutaneous Emphysema: A Case of Sudden-Onset Chest Pain in an Adolescent Vaper

**DOI:** 10.51894/001c.164420

**Published:** 2026-08-01

**Authors:** Alexander Almanza, Igor D. Middlebrook, Evan Bruce

**Affiliations:** 1 University of Michigan Health - Sparrow

## 57

### Case Description

A 17-year-old male with a history of asthma, nicotine (vaping), and marijuana use presented to the emergency department (ED) with dyspnea in the setting of 2-3 days of worsening cough and congestion. The night prior, he developed sudden-onset pleuritic anterior chest pain, described as the “worst pain of his life.” Upon awakening, he was dyspneic and his pain had extended throughout his neck. He denied fever, nausea, vomiting, or recent strenuous activity. On presentation, he appeared to be in pain. Vitals were stable. Physical examination revealed tachypnea, decreased air movement, non-exudative posterior oropharyngeal erythema, Hamman’s sign, and palpable subcutaneous emphysema. Labs were notable for leukocytosis of 22.5 with neutrophilic predominance. Group A strep and COVID/Flu/RSV swabs were negative. Cardiovascular, neurological, and abdominal exams were unremarkable. Chest X-ray showed bilateral subcutaneous emphysema and pneumomediastinum without pneumothorax. CT of the neck and chest confirmed severe subcutaneous emphysema tracking bilaterally from the neck into the thorax and left flank, moderate-to-severe pneumomediastinum, and pneumorrhachis of the thoracic and cervical spine surrounding the thecal sac. The patient was admitted to PICU after treatment in the ED with analgesics, dexamethasone, and DuoNebs. He tested positive for rhinovirus/enterovirus. Fluoroscopic esophagram was unremarkable. ENT and cardiothoracic surgery recommended conservative management with oxygen, antibiotics, albuterol, atrovent, analgesics, and smoking cessation. He was discharged home following radiographic and symptomatic improvement.

### Discussion

Spontaneous pneumomediastinum (SPM) is a rare condition with a wide range of incidence ranging from 1 in 800 to 1 in 42,000 adult and pediatric patients. It is often seen in young males with underlying pulmonary risk factors. Typically, it results from alveolar trauma secondary to increased intrathoracic pressure from coughing, vomiting, or strenuous activity. This case highlights a rare form of SPM tracking into the spinal canal with no apparent source as determined per imaging. Given this patient’s history of asthma and smoking, it highlights the possible risks of inhalational exposure and the importance of considering SPM in a differential diagnosis in adolescent patients with risk factors.

